# Unloved, paraphyletic or misplaced: new genera and species of small to minute lucinid bivalves and their relationships (Bivalvia, Lucinidae)

**DOI:** 10.3897/zookeys.899.47070

**Published:** 2019-12-12

**Authors:** John D. Taylor, Emily A. Glover

**Affiliations:** 1 Department of Life Sciences, The Natural History Museum, London, SW7 5BD, UK The Natural History Museum London United Kingdom

**Keywords:** bivalves, chemosymbiosis, taxonomy, Indo-West Pacific, South Atlantic

## Abstract

Species identified as *Pillucina* are paraphyletic in molecular analyses and a new generic name, *Rugalucina*, is introduced for a complex of three similar species *Rugalucina angela* from the northern Indian Ocean and Red Sea, *R. vietnamica* from South East Asia, and *R. munda* from northern and north eastern Australia. *Lucina concinna* from the Red Sea, previously synonymised with *P. vietnamica/angela* is recognised as a *Rugalucina*-like species but with a very short anterior adductor scar. *Divaricella cypselis* from Karachi is similarly now recognised as a distinct species, probably related to *Rugalucina* but with oblique commarginal sculpture and a short adductor scar. A group of minute Indo-West Pacific lucinids with highly unusual multi-cuspate lateral teeth and previously classified as *Pillucina* are separated under a new genus *Pusillolucina***gen. nov.**, with the description of three new species *P. arabica*, *P. africana*, and *P. biritika* from the Arabian Gulf, Mozambique, and Madagascar. Finally, a new genus, *Notocina*, is introduced for the small southern Atlantic species, *Epicodakia falklandica*, shown in molecular analyses to be misplaced at subfamily level and now classified in Lucininae and not Codakiinae with *Epicodakia*.

## Introduction

Within the chemosymbiotic bivalve family Lucinidae, genera in the *Loripes* group have been identified as monophyletic (Taylor et al. 2011, 2016) and characterised by a distinctive internally inset ligament. In the eastern Atlantic and Mediterranean *Loripes orbiculatus* is recognised as the most abundant lucinid in shallow water seagrass habitats and its important role in ecosystem function has been much studied (Johnson et al. 2002; Heide et al. 2012; Rossi et al. 2013; Geest et al. 2014). Other genera within the *Loripes* group include *Lucinella* and *Keletistes* in the Atlantic and from the Indo-West Pacific *Pillucina*, *Wallucina*, and *Chavania*. Of these, *Pillucina* is the most diverse with eleven currently recognised species (Glover and Taylor 2001; 2016) distributed from the Red Sea to the Hawaiian Islands. Some species have been recorded with high population densities in seagrass and peri-mangrove habitats (Nakaoka et al. 2002; Uede and Takahashi 2008, Meyer et al. 2008; Rattanachot and Prathep 2015). However, recent and ongoing molecular analyses indicate that *Pillucina* is paraphyletic with *P. vietnamica* Zorina, 1978 and *P. pusilla* Glover & Taylor, 2016 aligning distantly from five other *Pillucin*a species (Taylor et al. 2011, 2016, Glover et al. 2016). Moreover, it is also now apparent that the reportedly widespread *Pillucina vietnamica* is a complex of three similar species with distinct distributional ranges. In view of the paraphyly and the separate status of the *Pillucina vietnamica* species group we propose a new genus and a reappraisal of the constituent species including two names resurrected from synonymy and recognition of an early available name from Australia.

The other lucinid separated by paraphyly and not closely related to the other *Pillucina* species is the minute *Pillucina pusilla* from the Philippines that possesses distinctive multi-cuspate lateral teeth. This extremely unusual character was first recognised in *Pillucina denticula* Glover & Taylor, 2001 from near Durban, South Africa. Recently, we have identified herein other species of minute lucinids with similar dentition from the Arabian Gulf, Mozambique and northern and southern Madagascar. Species with this dentition form a morphologically distinct clade that we recognise with a new generic name.

Additionally, amongst these small lucinids it is apparent that the southern Atlantic species *Epicodakia falklandica* Dell, 1964 is misclassified because molecular results (Taylor et al. 2011, 2016) place it in the large subfamily Lucininae rather than Codakiinae with other *Epicodakia* species. We review the morphology and relationships of this species and place it in a new genus.

During the last 20 years there has been a marked proliferation of generic categories within Lucinidae building on and revising the prior classifications of Chavan (1969) and Bretsky (1976). This is a reflection of increased taxonomic activity following the discovery of chemosymbiosis but also the clear indication from molecular results and more detailed morphological studies that a number of existing genera are paraphyletic or polyphyletic, with some species misclassified even at subfamily level. At species level increased sampling effort, with closer attention to smaller sieve size fractions, has revealed an unexpected range of minute lucinids and this, coupled with greater attention to museum collections and type material, has highlighted previously neglected species.

### Institutional abbreviations

**AMS** Australian Museum, Sydney

**BAS** British Antarctic Survey

**MCG** Museo Civico, Genoa

**MNHN** Muséum national d’Histoire naturelle, Paris

**NHMUK** Natural History Museum, London

**NMSA** KwaZulu-Natal Museum, Pietermaritzburg South Africa

**NMV** National Museum of Victoria, Melbourne

**NMW** National Museum of Wales

**UMZC** University Museum of Zoology, Cambridge, UK

**WAM** Western Australian Museum, Perth

**ZISP** Zoological Institute, St Petersburg, Russia

### Other abbreviations

**IWP** Indo-West Pacific

**L** shell length

**LV** left valve

**P1** protoconch 1 length

**P2** protoconch 2 length

**RV** right valve

**SEM** scanning electron microscopy

**sh** complete shell both valves

**v** single valve

## Systematics

### Family Lucinidae Fleming, 1828

#### Subfamily Lucininae Fleming, 1828

##### Rugalucina gen. nov.

Taxon classificationAnimaliaLucinidaLucinidae

8A063CD8-9A35-5374-B254-2B91245A9672

http://zoobank.org/09C8B8AD-4DF9-48A5-8D17-EACA3B276B11

###### Type species.

Lucina (Codakia) angela Melvill, 1899. Here designated.

###### Diagnosis.

Small L to 15 mm, sub-circular, sculpture of fine, commarginal lamellae crossed by strong radial ribs more prominent to anterior and posterior, with overall crinkled appearance, ligament largely internal, obliquely inset, anterior adductor muscle scar ventrally detached from pallial line for half of length, inner shell margin crenulate.

###### Etymology.

From Latin *ruga* for wrinkle or crease and *Lucina*, feminine.

###### Included species.

Lucina (Codakia) angela Melvill, 1899, *Pillucina vietnamica* Zorina, 1978, Lucina (Codakia) munda A. Adams, 1856. Tentatively included: *Divaricella cypselis* Melvill, 1918 and *Lucina concinna* H. Adams, 1871.

###### Comparison with other genera.

*Rugalucina* is part of the broader *Loripes* group within the Lucininae, all having an obliquely inset internal ligament. Other genera within the group are *Pillucina*, *PusillolucinaWallucina*, *Lucinella*, *Chavania*, and *Keletistes*. Of these only *Pillucina*, *Pusillolucina*, and *Rugalucina* have prominent radial sculpture. In shell characters *Rugalucina* differs from *Pillucina* in the more strongly divergent radial sculpture, the longer anterior adductor muscle scar and the more coarsely crenulate inner shell margin.

**Figure 1.**
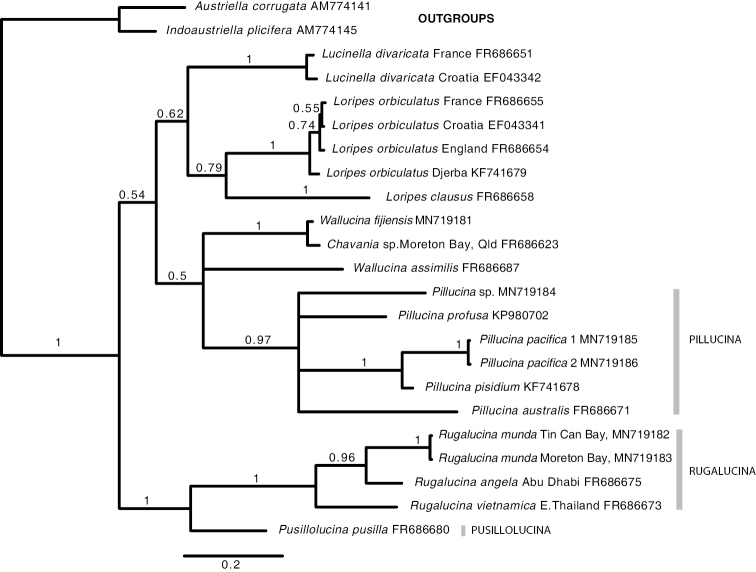
Single gene tree for the *Loripes* group of genera and species based on cytochrome b gene sequences, using Bayesian inference as implemented by MrBayes. Support values are posterior probabilities. Methods as in Taylor et al. (2011, 2016). GenBank numbers are attached to names. Newly sequenced species: *Wallucina fijiensis* Kavieng, Papua New Guinea (MNHN IM- IM-2013-54066), *Pillucina pacifica*, Kavieng, KAV 1 (MNHN IM- IM-2013-54743), KAV 2 (MNHN IM-2013-51690), *Pillucina* sp. Kavieng, PNG (MNHN IM-2013-54590), *Rugalucina munda* North Stradbroke Island, Moreton Bay, Qld, Australia (NHMUK 20191069), *R. munda* Tin Can Bay, Qld, Australia (NHMUK 20191070).

In molecular analyses members of the *Loripes* group form a monophyletic subclade of Lucininae. Seven species of putative *Pillucina* have been included in analyses (Taylor et al. 2016), *P. pisidium*, *P. symbolica*, *P. australis*, *P. profusa*, *P. pacifica*, ‘P.’ pusilla, and ‘P.’ vietnamica, the latter from Thailand, Abu Dhabi, and Queensland. Although the type species *P. hawaiiensis* has not yet been included, *P. pacifica* is similar in morphology and could be regarded as a proxy. The *Pillucina* species are not monophyletic in published trees or in the mitochondrial cytochrome b gene tree (Fig. 1), with those identified as *P. vietnamica* and *P. pusilla* separated from the other species and now classified herein into two new genera with distinct morphological characters. Note: we use the cyt b gene in the analysis of the *Loripes* group shown in Fig. 1 as this best reflects species level relationships within clades of the subfamily Lucininae as shown by previous analyses using three genes (Taylor et al. 2011, 2016).

Previously, we regarded *Pillucina vietnamica* as a wide-ranging species in the northern Indian Ocean through south east Asia to southern China (Glover and Taylor 2001), the name replacing the earlier but preoccupied *Lucina fischeriana* Issel, 1869, with *P. angela* as an possible phenotype from the northern Arabian Sea. Although more comprehensive sampling across the whole range is desirable, evidence from analysis of the cytochrome b gene (Fig. 1) shows that specimens from Tin Can Bay and Moreton Bay, Qld, Australia differ from samples from Kungkraben Bay, Thailand and Abu Dhabi supporting a species level separation. Our evidence suggests that *Pillucina* ‘*vietnamica*’ from Arabia, Thailand and Queensland are distinct. After further study of type material, we use the name *R. angela* for the northern Indian Ocean species, *P. vietnamica* for the southeast Asian species, and *P. munda* for the northern Australian species.

*Lucina concinna* H. Adams, 1871 previously placed in the synonymy of *P. vietnamica* by Glover and Taylor (2001) is now recognised as having distinct morphological characters and renamed because the original species name is preoccupied. We also resurrect *Divaricella cypselis* Melvill, 1918 from the synonymy of *R. angela* and recognise it as another morphologically distinct species, from the Arabian Sea and southern India.

##### Rugalucina angela

Taxon classificationAnimaliaLucinidaLucinidae

(Melvill, 1899)

7FBF93FC-9945-5711-A759-808852ACCB2E

Figs 2 , 3

Lucina fischeriana Issel, 1869: 83–84, pl. 1, fig. 8 (non L. fischeriana d’Orbigny, 1845: Jurassic fossil).
Lucina (Codakia) angela Melvill, 1899: 98, pl. 2, fig. 8.
Loripes fischeriana : Lamy 1916: 151.
Pillucina fischeriana : Oliver 1992: 98, pl. 20, fig. 4.
Pillucina fischeriana : Oliver 1995: 236, fig. 1026.
Pillucina angela : Oliver 1995: 236, fig. 1025.
Pillucina vietnamica (part): Glover and Taylor 2001: 273.
Pillucina angela : Glover and Taylor 2001: 279, figs 9 h, i.
Pillucina angela (part): Huber 2015: 422, figs p. 76.

###### Type material.

*Lucina fischeriana* 5 syntypes (MCG), type locality: Suez, Egypt.

*L. angela* two syntypes NHMUK1899.12.18.20-21; L 7.9 mm and 6.1 mm; 1 syntype NMW 1955.158.684.

###### Type locality.

Gwadur, Pakistan, 8 fathoms (15 m).

###### Description.

Small (L to 15 mm), subcircular, inflated. Colour white, yellow or orange. Waxy appearance. Sculpture of strong diverging radial ribs, broader and more widely spaced to the anterior and posterior. Ribs crossed by fine, closely spaced, commarginal lamellae which curve over ribs producing a roughly scabrous appearance. Central parts of shell generally without ribs. Lunule large, broadly lanceolate, smooth. Ligament internal, obliquely inset. Hinge: RV with single large cardinal tooth and short anterior and posterior lateral teeth, LV with two cardinal teeth, lateral teeth consisting of small sockets. Anterior adductor scar narrow, elongate, detached from pallial line for ca. half of length. Pallial line irregularly lobate, or slightly divided. Inner shell margin coarsely crenulate to anterior and posterior.

###### Distribution.

**Red Sea**: Great Bitter Lake (Hoffman et al. 2006), Suez Canal, El Ballah (NHMUK 1950.11.10.1), Suez (NHMUK 1968.5.29.2), Safaga, Dongonab Bay (NHMUK), Oreste Point, Yemen (Dekker colln), Aden (NHMUK 1963340). **Arabian Gulf**: Kuwait (NHMUK), Tarut Bay, Qatar (NHMUK), Abu Dhabi (NHMUK), Ras al Khaimah (NHMUK). **Arabian Sea**: Khor Kalba, Sharjah (NHMUK), Karachi, dredged (NHMUK 1953.1.30.85). Oman: Masirah Island (NHMUK). **Indian Ocean**: South India: Chennai (Madras) (NHMUK 1953.1.30.169-73), Krusadai Island (NHMUK 1953.1.30.110), Kundugal Point, Krusadai Island (NHMUK1953.1.30.175-181), Tuticorin (NHMUK 1953.1.30.99-101). Sri Lanka: Trincomalee (NHMUK 1910.9.28.175-178).

*Rugalucina angela* (as *Pillucina vietnamica*) is recorded as an invasive species off Israel in the eastern Mediterranean (Steger et al. 2018).

###### Remarks.

*Rugalucina angela* shows variation in shell morphology between various localities around the Arabian Peninsula and Red Sea. For example, shells from the Arabian Gulf are usually smaller, while those from the northern Red Sea as on Gulf of Suez shores are generally larger, and the marginal crenulations stronger. We regard these differences as ecophenotypic probably associated with the extreme environmental conditions such as the very high salinities experienced in the southern Arabian Gulf, usually more than 40 psu but in shallow lagoons as high as 52–55 psu (Price 1982) and approximately 40–46 psu at Safaga in the northern Red Sea (Zuschin and Oliver 2003) and probably higher for the habitats occupied by *Rugalucina*.

*Rugalucina angela* is closely similar in shell morphology to *R. vietnamica* and *R. munda*. The differences are subtle; externally they share diverging radial ribs that are stronger to anterior and posterior, and the fine commarginal lamellae. *Rugalucina angela* has a shorter anterior dorsal area, larger hinge plate and teeth, and a slightly more divergent anterior adductor scar. *Rugalucina vietnamica* is higher, with a longer anterior dorsal area. *Rugalucina munda* is similar but the radial sculpture is much less pronounced with finer margin denticulations and subdued commarginal sculpture.

**Figure 2.**
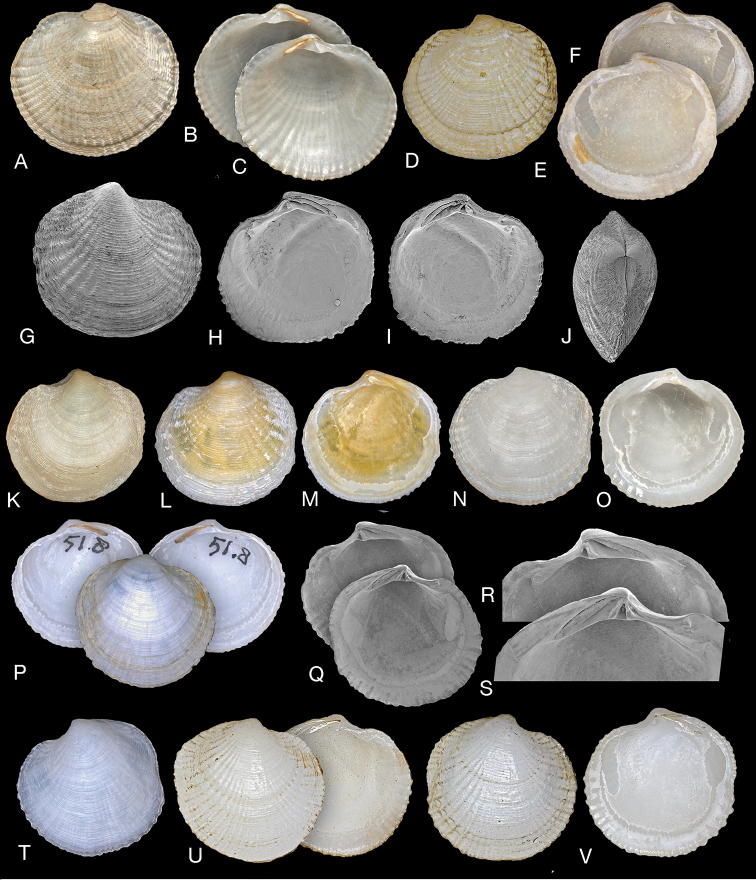
*Rugalucina angela* (Melvill, 1899). **A–C** Syntype of Lucina (Codakia) angela Melvill, 1899 (NHMUK 1899.12.18.20), exterior of left valve and interiors of right and left valves. Gwadur, Pakistan, L 8.1mm **D–F**L. (C.) angela syntype (NHMUK 1899.12.18.20), exterior of left valve and interiors of right and left valves, L 6.1 mm **G–M***Rugalucina angela* Ras al Khaimah, Arabian Gulf, (NHMUK 20191071) **G** exterior SEM of right valve, L 5.0 mm **H, I** interior SEM of right and left valves, L 7.7 mm **J** dorsal view, L 5.9 mm **K** exterior of left valve, L 7.9 mm **L, M** exterior of left valve, interior of right valve, L 7.6 mm **N, O***R. angela* exterior and interior of left valve, Gulf of Suez (NHMUK1868.5.29.2), L 13.7 mm **P***R. angela* exterior of right valve and interior of right and left valves, Egypt, 7km south of Hurgada, H Dekker colln 4569, L 12.8 mm **Q** interior of left and right valves, Egypt, Port Safaga, H Dekker colln 3263, L 9.4 mm **R, S** detail of hinge teeth of **Q**. **T***R. angela* exterior of left valve. Red Sea, Yemen, Orestes Point, N of Midi, H Dekker colln 4553, L 9.8 mm **U***R. angela* exterior of right and interior of left valves, Aden (NHMUK 1963340), L 10.6 mm **V***R. angela* exterior and interior of right valve, Krusadai, India, (NHMUK 1953.1.30.69-76), L 8.4 mm.

**Figure 3.**
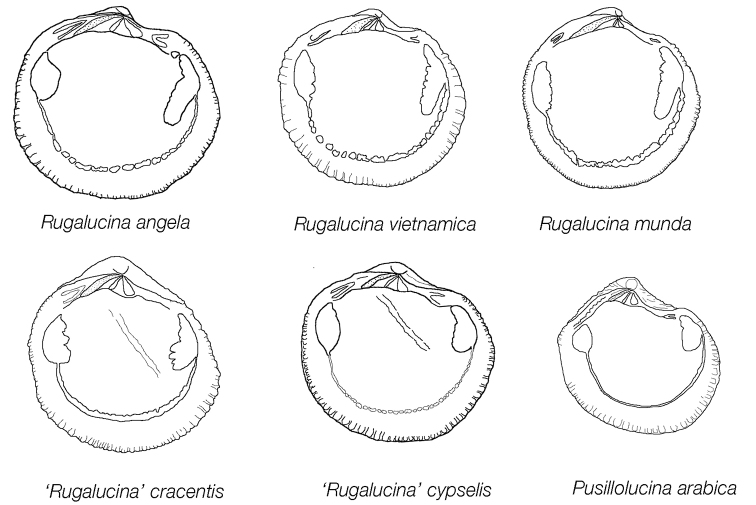
Internal drawings of left valves of *Rugalucina* and *Pusillolucina* species. Not to scale.

##### Rugalucina vietnamica

Taxon classificationAnimaliaLucinidaLucinidae

(Zorina, 1978)

375FA719-4F6C-545F-B380-1C7765697ED0

Figs 3 , 4

Pillucina vietnamica Zorina, 1978:195, figs 3 & 6).
Pillucina vietnamica : Lutaenko 2000: 383, pl. 1, figs 1, 2; pl. 3, fig. 1.
Pillucina vietnamica (part): Glover and Taylor 2001: 273, figs 7a-g.
Pillucina vietnamica : Glover et al. 2016: 553, fig. H, A-M.
Pillucina angela (Melvill, 1899) (part): Huber 2015: 422, figs p. 76.

###### Type material.

*Pillucina vietnamica* syntypes (ZISP), 13 shells and 1 valve, L 5.5–8.9 mm.

###### Type locality.

Intertidal, south coast of Hainan, China.

###### Description.

Small (L to 10 mm), sub-circular, longer than high, posteriorly slightly truncate, moderately inflated. Shell white, slightly translucent and waxy in appearance. Sculpture of many, fine, low, commarginal lamellae and low radial ribs which are broader and more prominent towards the anterior and posterior. Radial ribs are conspicuously fluted where commarginal lamellae cross giving a crinkled appearance. Lunule elongate, lanceolate and impressed, slightly asymmetrical. Ligament internal, short, situated on a broadly triangular resilifer. Hinge: right valve with single, cardinal tooth, anterior and posterior lateral teeth small, posterior tooth elongate. Left valve with two narrow cardinal teeth, a small anterior lateral socket and posterior lateral narrow socket. Anterior adductor muscle scar medium-long, detached for ca. 50 % of length. Posterior scar ovate. Pallial line entire, sometimes partially discontinuous or irregularly lobate. Shell margin crenulate, with crenulations coarser towards anterior and posterior.

###### Distribution.

**Singapore**: Pulau Semakau (NHMUK), Seringat Bay (NHMUK). **Malaysia**: Langkawi (AMS). **Western Thailand**: Ao Bang Ben, Kapoe (NHMUK), Ban Bang Ben (NHMUK), Tung Nam Dan, Phang Nga Province (NHMUK), **Eastern Thailand**: Kungkraben Bay (NHMUK). **Cambodia**: 5 km E of port, Sihanoukville (NHMUK). **Vietnam**: Dam Bay near Nha Trang (Zvonareva et al. 2019). **China**: Hainan (MNHN), Hong Kong (NHMUK), Daya Bay (NHMUK).

###### Habitat.

**Figure 4.**
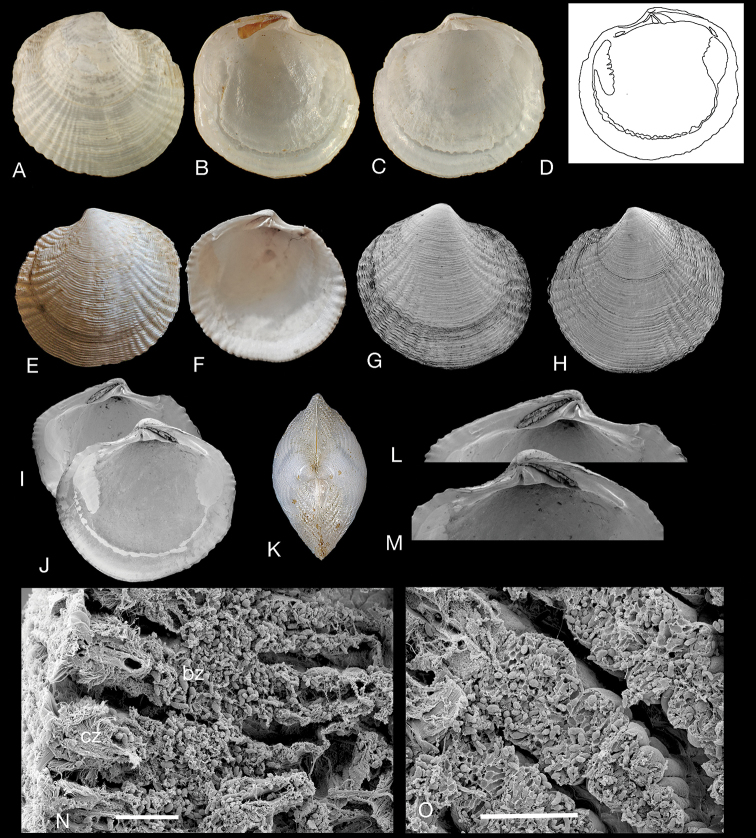
*Rugalucina vietnamica* (Zorina, 1978). **A–C***Pillucina vietnamica* Zorina, 1978 syntypes (ZISP), exterior of right valve and interior of left and right valves, Hainan, China, L 5.5–8.9 mm **D** internal drawing of **C. E, F***R. vietnamica* Palau Semaku, Singapore (NHMUK 20150057), L 8.5 mm **G–L***R. vietnamica* Kungkraben Bay, eastern Thailand (NHMUK 20191072) **G** exterior of right valve, L 7.0 mm. **H** exterior of left valve, L 7.0 mm **I, J** interior of left and right valves, L 7.7 mm **K** dorsal view L 5.6 mm **L, M** detail of hinge of **I** and **J**. **N** Section through gill of *Rugalucina vietnamica* Kungkraben Bay, Thailand showing filaments with ciliary (cz) and bacteriocyte zones (bz). Critical-point dried preparation **O** detail showing abundant bacterial symbionts in bacteriocytes. Scale bars: 20 µm (**N**); 10 µm (**O**).

Intertidal to shallow sub-tidal seagrass and muddy habitats. Recorded at high densities in muddy seagrass and mangrove fringe habitats of eastern and western Thailand (Meyer et al. 2008; Rattanachot and Prathep 2016). Gill structure and symbiotic bacteria are illustrated in Fig. 4N, O.

###### Remarks.

Although we formerly recorded *Rugalucina vietnamica* with a broad longitudinal range from the northern Red Sea to southern China (Glover and Taylor 2001) we now regard it as having a narrower range along the continental margin of south eastern Asia with few records from islands. Despite intensive sampling by the MNHN Paris Expeditions around Panglao, Philippines (2004, 2005) and Papua New Guinea (2012, 2014) no *R. vietnamica* were recorded (Glover and Taylor 2016 and unpublished data).

##### Rugalucina munda

Taxon classificationAnimaliaLucinidaLucinidae

(A. Adams, 1856)

5C3E165B-9DAD-599F-8256-94192C021543

Figs 3 , 5

Lucina (Codakia) munda A. Adams, 1856: 225 not figured.

###### Type material.

**Figure 5.**
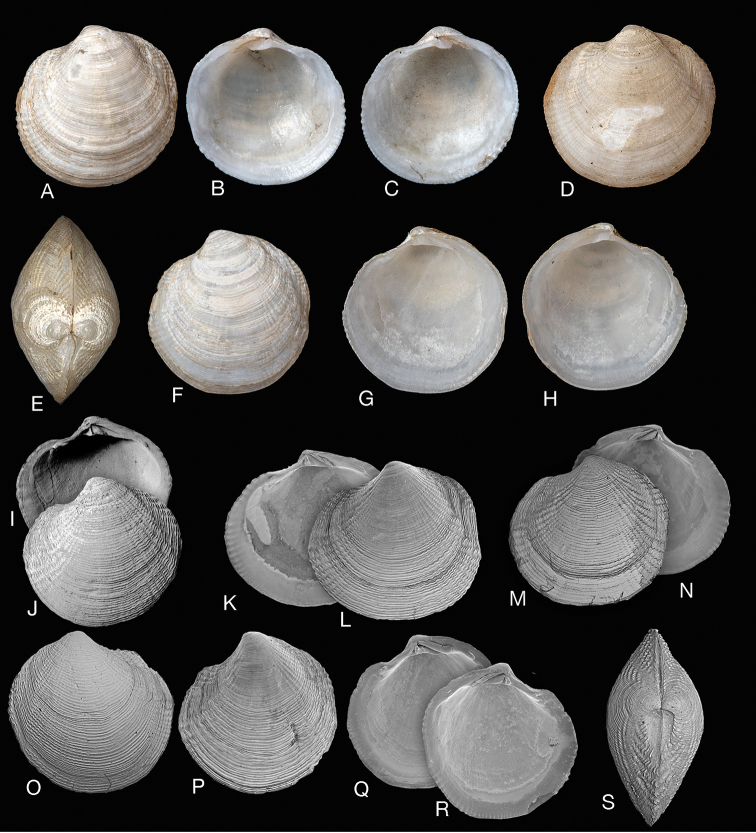
*Rugalucina munda* (Adams 1856). **A–H** Syntypes Lucina (Codakia) munda A. Adams, 1856 (NHMUK 20140004) Moreton Bay, Queensland, Australia **A–C** exterior and interior of left and right valves, syntype A, L 11.3 mm **D, E** exterior of left valve and dorsal view, syntype B, L 11.2 mm **F–H** exterior of left valve and interior of left and right valves, syntype C, L.10.4 mm **I, J***Rugalucina munda* exterior of left valve and interior of right valve, Redland Bay, Moreton Bay, Queensland (NHMUK 20191073), L 6.8 mm **K–N***R. munda* Tin Can Bay, Queensland (NHMUK 20191074) **K** Exterior left valve, L 6.9 mm **L** interior of right valve, L 6.3 mm **M** interior of left valve, L 5.5 mm **N** exterior of left valve, L 6.7 mm **O–S***R. munda* Port Douglas, Queensland (NHMUK 20191085) **O** exterior of right valve, L 6.5 mm **P** exterior of left valve, L 4.8 mm **Q, R** interior of right and left valves, L 7.2 mm **S** dorsal view, L 5.5 mm.

***Syntypes***: 3 whole shells NHMUK 20140004. H. Cuming colln, collected by Mr Strange. Shell lengths 11.3 mm, 11.2 mm, 10.4 mm. (Fig. 5A–H).

###### Type locality.

Moreton Bay, Queensland, Australia.

###### Material examined.

**Australia**: Western Australia: Parry Harbour, Kimberley (WAM). Broome Bay (WAM). Northern Territory: Darwin, East Point. Gove (NMV), Groote Eylandt G. of Carpentaria (AMS). Friday Island, Torres Strait (AMS). Queensland: Somerset, Cape York (AMS), Port Douglas (16°28'48"S, 145°27'41"E) seaward edge of mangroves (NHMUK). Buchan Point, Cairns (AMS), Port Denison, Bowen (AMS), Seaforth, Mackay (AMS), Yeppoon (AMS), Tin Can Bay, Snapper Point (25°54'12.18"S, 153°00'58"E), mangroves, muddy sand. Moreton Bay, Redland Bay (27°37'03"S, 153°19'06"E), muddy seagrass, 1 m. North Stradbroke Island, near Dunwich, seagrass (27°29'44"S, 153°23'57"E).

###### Description.

Small, L to 11 mm, sub-circular, posteriorly slightly truncate in juveniles, shallow posterior sulcus, moderately inflated. Shell colour white, internally often yellowish. Sculpture of fine, low commarginal lamellae crossed by low radial ribs which are broader and more prominent towards the anterior and posterior. Radial ribs are slightly fluted where crossed by commarginal lamellae. Lunule elongate, lanceolate, slightly asymmetrical. Ligament internal, short, situated on a broadly triangular resilifer. Hinge: right valve with single, narrow, cardinal tooth, anterior and posterior lateral teeth small; left valve with two narrow cardinal teeth, small anterior lateral and posterior lateral teeth. Anterior adductor muscle scar medium length, detached for ca. 50 % of length. Posterior scar ovate. Pallial line entire, irregularly lobate. Shell margin finely crenulate, with crenulations coarser towards anterior and posterior.

###### Distribution.

Northern and north eastern Australia.

###### Habitat.

Intertidal and shallow subtidal muddy sand, nearshore seagrass and mangrove fringe.

###### Remarks.

*Rugalucina munda* is closely similar in shell morphology to *R. vietnamica* and *R. angela* and we previously confounded the species (Glover and Taylor 2001). In shell morphology the differences are subtle; *R. munda* is more ovoid, the radial ribs are finer, and the inner shell margin is less coarsely crenulated compared to *R. angela* and *R. vietnamica*. Furthermore, Glover and Taylor (2001: fig. 16) recorded a distributional gap between Australian and Asian records of ‘*P. vietnamica*’. Features of the anatomy and ctenidial bacteria of *R. munda* from Port Douglas, Qld were described by Glover and Taylor (2001, as *Pillucina vietnamica*).

Hedley (1913: 267) recommended that the name *Lucina munda* be rejected as unrecognisable because at that time type material had not been identified and the original description ambiguous. Later, because the incorrect type material had been isolated in the NHMUK collection the name *Lucina munda* had been regarded (e.g., Lamprell and Whitehead 1992) as a synonym of *Ctena bella* (Conrad, 1837). More recent recognition of the original type material (JDT & EAG) showed that Lucina (Codakia) munda (syntypes figured here for the first time from the Cuming collection and collected in Moreton Bay by Mr Strange) is a species similar to *Rugalucina vietnamica* and *R. angela*. In his description Adams (1856) highlights the dichotomously radiating ribs and the radially grooved inner shell margin and the yellowish shell interior. *Rugalucina munda* is thus the earliest name for the Australian species.

##### *Rugalucina* sensu lato

Here we include two species from the northern Indian Ocean and Red Sea that are similar externally to *Rugalucina vietnamica* but differ from it and each other in shell sculpture, hinge, and muscle scar characters. No molecular material is available for firmer placement of either species.

###### ‘Rugalucina’ cypselis

Taxon classificationAnimaliaLucinidaLucinidae

(Melvill, 1918)

07F2635D-C673-5922-AC75-4BBF54EE315E

Figs 3 , 6

Divaricella cypselis Melvill, 1918: 156, pl. 5, fig. 33.

####### Type material.

*D. cypselis* holotype NHMUK 1921.1.28.42. sh, L 5.2 mm.

####### Type locality.

Karachi, Pakistan, 20–30 fathoms (36–55 m).

####### Other material examined.

**Pakistan**: Karachi, Winckworth collection (NHMUK 20191075) 55 sh, 52 v. **India**: dredged Chennai (Madras), Winckworth Collection (NHMUK 1958.1.30.45) 1 sh. Chennai (Madras) (NHMUK 1953.1.30.169-73 part) 5 v.

####### Description.

**Figure 6.**
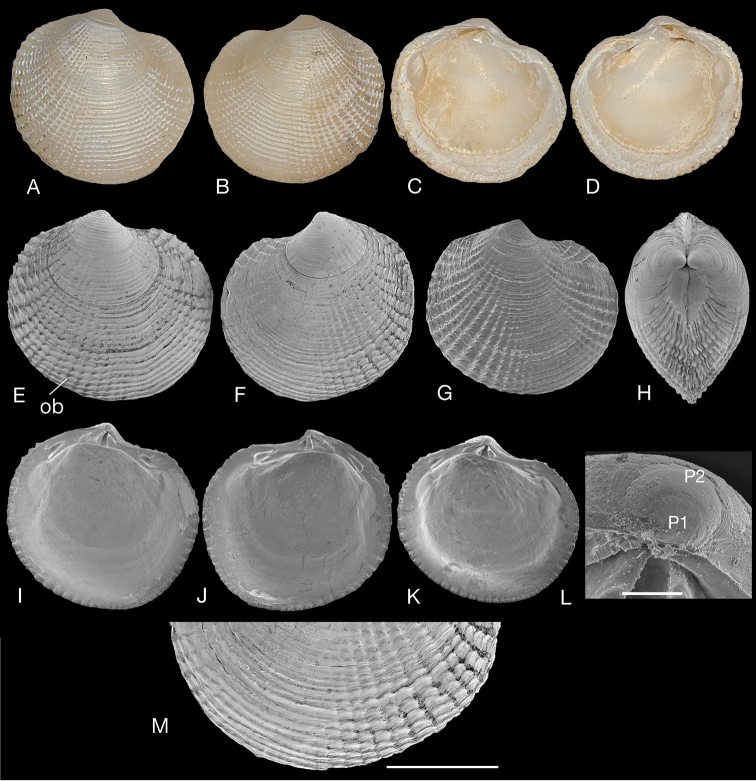
‘Rugalucina’ cypselis (Melvill, 1918). **A–D** Holotype *Divaricella cypselis* Melvill, 1918 (NHMUK 1921. 1. 28. 92), Karachi, L 5.2 mm **E–L** ‘Rugalucina’ cypselis Karachi (NHMUK 20191075) Winckworth collection **E** right valve, L 3.1 mm, ob – oblique commarginal lamellae **F** left valve, L 3.0 mm **G** right valve, L 3.2 mm **H** dorsal view, L 1.9 mm **I** interior left valve, L 3.2 mm **J** interior right valve, L 3.2 mm **K** interior left valve, L 3.0 mm **L** protoconch **M** detail of ventral margin (**F**) showing obliquely aligned commarginal lamellae. Scale bar: 100 µm (**L**); 1 mm (**M**).

Small (L to 5.2 mm), sub-circular, inflated. Colour white or yellowish. Sculpture of diverging, curved, radial ribs prominent to anterior and posterior but are subdued or absent in middle parts of shell. Ribs crossed by closely spaced, narrow, low, commarginal lamellae that are aligned obliquely to the ventral shell margin (Fig. 6M). Radial ribs with small scales where crossed by commarginal lamellae. Early parts of shell relatively smooth. Protoconch P1 ca 88 µm, P2 ca 170 µm with growth increments (Fig. 6L). Lunule broadly lanceolate, smooth. Ligament largely internal, short, set on oblique resilifer. Hinge: right valve with single cardinal tooth and prominent anterior and posterior lateral teeth; left valve with two cardinal teeth, the anterior larger and sockets for anterior and posterior lateral teeth. Anterior adductor muscle scar, short, broad, ventrally detached from pallial line for ca. 15 % of length, posterior scar ovoid. Pallial blood vessel scar visible. Pallial line discontinuous in small blocks. Shell margin dentate, coarser towards anterior and posterior.

####### Distribution.

Known from the northern Arabian Sea and southern India. Probably more abundant in the northern Indian Ocean but unrecognised.

####### Remarks.

**Figure 7.**
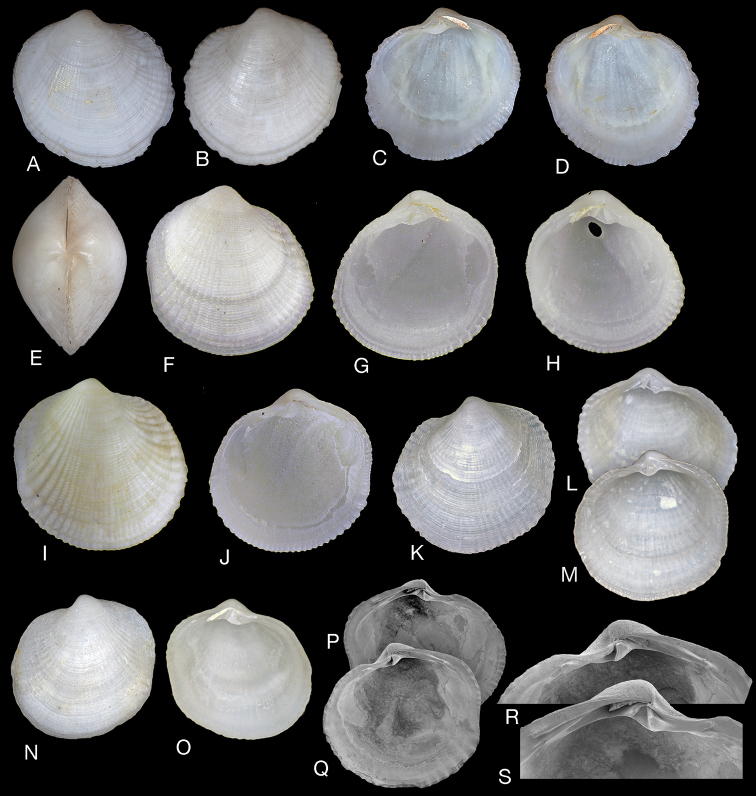
‘Rugalucina’ cracentis sp. nov. **A–E***Lucina concinna* Holotype (ZMC I.100470) Gulf of Suez, exterior and interior of right and left valves and dorsal view, L 9.2 mm **F–H** ‘*Rugalucina cracentis*’ exterior of right valve and interior of right and left valves Egypt, Dahab, Gulf of Aqaba, Red Sea. H. Blatterer colln, L 6.7 mm **I, J***R. cracentis* exterior and interior of right valve Dahab, Gulf of Aqaba, Red Sea, H. Blatterer colln, L 8.5 mm **K, L***R cracentis* exterior and interior of left valve Egypt, 4 km north of Port Safaga, H. Dekker colln 6930, L 8.3 mm **M** interior of right valve, L 8.8 mm **N, O** exterior and interior left valve Egypt, 4 km north of Port Safaga, H. Dekker colln 6930, L 7.1 mm **P, Q** interiors of right and left valves, H. Dekker colln 6930, L 8.7 mm **R, S** hinge details of **P** and **Q**.

Dekker and Goud (1995: 6) excluded *D. cypselis* from *Divaricella* more correctly suggesting placement in *Pillucina*. Whereas previously we included *Divaricella cypselis* in the synonymy of *Pillucina angela* because of the similarity of external sculpture (Glover and Taylor 2001) we now recognise it as a morphologically distinct species. The holotype and other specimens from the Karachi area all have a very short anterior adductor muscle scar (Figs 3, 7I–K) that is barely detached from the pallial line compared with the longer and ventrally detached scar of *Rugalucina angela*, *R. vietnamica* and *R. munda*. It also has a distinctive sculpture of oblique commarginal lamellae set at an angle to the ventral shell margin that is not seen in other *Rugalucina*. Similar radial ribs and dentate inner shell margin are present in *Pusillolucina* species, but they have multi-cuspate posterior lateral teeth and lack the oblique commarginal sculpture.

The obliquely inset internal ligament indicates placement in the *Loripes* group of genera and while the external sculpture resembles *Rugalucina* in radial ribbing the obliquely aligned commarginal lamellae are similar to that seen in *Lucinella divaricata* (Linnaeus, 1758) from the eastern Atlantic but that species lacks any radial ribbing.

###### ‘Rugalucina’ cracentis sp. nov. (replacement name)

Taxon classificationAnimaliaLucinidaLucinidae



D5F21F2F-8C24-58AB-A7F4-9DDE68633E43

http://zoobank.org/1D3FC94A-5371-40EC-AB78-B85AF858A230

Figs 3 , 7

Lucina concinna H. Adams, 1871: 791, pl. 48, fig. 13 (non Lucina concinna Deshayes, 1857 an Eocene fossil).
Loripes concinnus : Lamy 1916: 15.
? Pillucina concinna: Oliver 1992: 98, pl 20, figs 5a, b.
Pillucina cypselis (Melvill, 1918): Dekker and Orlin 2000: 11.
Pillucina vietnamica : Zuschin and Oliver 2003: pl. 24, figs 24.8-24-13.

####### Type material.

Holotype of *Lucina concinna*UMZC I.100470 Gulf of Suez, Red Sea. L 9.2 mm.

####### Etymology.

*cracentis* Latin, genitive singular of *cracens* meaning neat, graceful. Adjective.

####### Diagnosis.

Ovoid shape, slightly higher than long, diverging radial ribs, ligament short, largely internal, hinge with ventral flexure, right valve with single large cardinal tooth, anterior adductor muscle scar short.

####### Description.

Small, L to 9 mm. ovoid, slightly higher than long (H/L 1.01), inflated, umbones prominent, rounded. Colour white or yellowish. Sculpture of diverging radial ribs, coarser and more widely spaced to anterior and posterior, ribs finer and more subdued in middle parts of shell. Ribs crossed by fine, low, closely spaced, commarginal lamellae. Lunule short, heart shaped. Ligament short, obliquely inset. Hinge line with ventral flexure (Fig. 7R, S), right valve with single, relatively large cardinal tooth and small anterior and posterior lateral teeth, left valve with two cardinal teeth the anterior larger and a central socket, small anterior and posterior lateral teeth. Anterior adductor muscle scar short with lobate posterior dorsal edge, ventrally detached from pallial line for 15% of length; posterior scar ovate. Pallial line largely entire, irregularly lobate. Pallial blood vessel trace visible. Inner shell margin crenulate, more coarsely to anterior and posterior.

####### Distribution.

**Red Sea**: Egypt: Gulf of Suez (ZMC), Port Safaga (Dekker colln), Ras Baghdadi (Dekker colln), Sharm el Naga (Dekker colln), Makadi Bay (Dekker colln), Gulf of Aqaba: Dahab (Blatterer colln), Yemen -al Durayhimi (Dekker colln), Aden (NHMUK 1902.12.30.749). **Arabian Gulf**: Kuwait (NHMUK), Saudi Arabia, Tarut Bay (NHMUK 20191076).

####### Remarks.

Dekker and Orlin (2000) used *Pillucina cypselis* as a synonym of the preoccupied *Lucina concinna* but, as shown above, the species differ in external morphology. Although Glover and Taylor (2001) included this species in the synonymy of *Pillucina vietnamica* (NW Indian Ocean forms now *Rugalucina angela*) we consider it distinct. Externally, it is similar to *Rugalucina angela* but differs in the ovoid shape, the finer radial ribs, the much shorter anterior adductor scar, the ventral flexure of the hinge line and large cardinal tooth in the right valve.

The ovoid shape and flexured hinge line with the large cardinal tooth in the right valve are features of *Pillucina* s. s. but *Pillucina* species usually have less prominent radial ribbing (Glover and Taylor 2001, 2016). Molecular data are needed to determine placement in either *Pillucina* or *Rugalucina*.

###### Pusillolucina gen. nov.

Taxon classificationAnimaliaLucinidaLucinidae

78CDE75D-63F4-5159-B2E2-1154E8DEA07A

http://zoobank.org/EE71DE26-9C58-44AE-9531-39C064CA866A

####### Type species.

*Pillucina pusilla* Glover & Taylor, 2016. Here designated. Philippines.

####### Diagnosis.

Very small, L to 3 mm, sub-circular, higher than long. Umbones prominent. Sculpture of thin commarginal lamellae, elevated to anterior and posterior, crossed by radial ribs that are more prominent to anterior and posterior. Lunule broadly lanceolate, concave. Ligament short, largely internal. Hinge: RV with single cardinal tooth, large anterior lateral tooth and a complex multi-cuspate posterior lateral tooth consisting of up to ten cusps; LV with two cardinal teeth, the anterior larger, posterior lateral tooth formed of sockets for projecting cusps of the right valve. Anterior adductor scar very short, barely detached from pallial line, pallial line irregularly discontinuous. Inner shell margin crenulate.

####### Etymology.

Derived from Latin *pusilla* meaning very small and *Lucina*. Feminine

####### Comparison with other genera.

*Pusillolucina* is similar in many external characters including shape and sculpture to some *Pillucina* (type species *Pillucina spaldingi* Pilsbry, 1921 = *Pillucina hawaiiensis* (Smith, 1885)). By comparison no *Pillucina* species possess the unusual multi-cuspate posterior dentition. Moreover, *Pusillolucina pusilla* is distinct in molecular analyses (Fig. 1 and Taylor et al. 2016) from most *Pillucina* including five common shallow water Indo-West Pacific species but aligns with ‘*Pillucina vietnamica*’. This latter is now placed in a separate genus, *Rugalucina* (see below), whose species are larger and, significantly, even in minute juveniles, lack the multi-cuspate posterior lateral teeth of *P. pusilla* and its congeners.

####### Included species.

*Pillucina pusilla*, *Pillucina denticula* Glover & Taylor, 2001, *Pusillolucina africana* sp. nov., *Pusillolucina arabica* sp. nov., *Pusillolucina biritika* sp. nov. These species differ mainly in characters of the dentition.

####### Distribution.

Indo-West Pacific, low intertidal to 70 m.

###### Pusillolucina pusilla

Taxon classificationAnimaliaLucinidaLucinidae

Glover & Taylor, 2016

3D41EB42-B7C5-5D5F-A5A2-8377E4061AFC

Figs 8 A–F

Pillucina pusilla Glover and Taylor 2016: 159, figs 41D, 44 A–L.

####### Type material.

***Holotype***: MNHN-IM-2000-26591. ***Paratypes***: 6 (MNHN-IM-2000-26592, IM-2009-10362). L 1.2–1.8 mm.

####### Type locality.

Philippines, Bohol Island, Manga, 9°41.1'N, 123°51.4'E, 3–4 m, mud, [PANGLAO 2004: stn S19].

####### Description.

Very small, glossy, L to 1.8 mm, sub-circular, higher than long, inflated. Umbones prominent. Sulci and dorsal areas poorly defined. Sculpture of thin commarginal lamellae elevated to anterior and posterior, with 17–25 low, rounded radial ribs, divaricate in anterior part of shell. Juvenile shell with elevated commarginal lamellae but no radial ribs. Microsculpture of fine growth increments only. Protoconch P1 +P 2 = 134 µm, P2 with numerous growth increments. Lunule broadly lanceolate, depressed, asymmetric, larger part in left valve. Ligament short, internal, oriented parallel to cardinal teeth. Hinge: right valve with single cardinal tooth, large anterior lateral tooth and posterior lateral tooth consisting of four or five cusps, left valve with two cardinal teeth, the anterior larger, anterior lateral socket, posterior lateral with sockets for cusps of the right valve. Anterior adductor scar very short, barely detached from pallial line, pallial line irregularly discontinuous. Inner shell margin coarsely crenulate.

**Figure 8.**
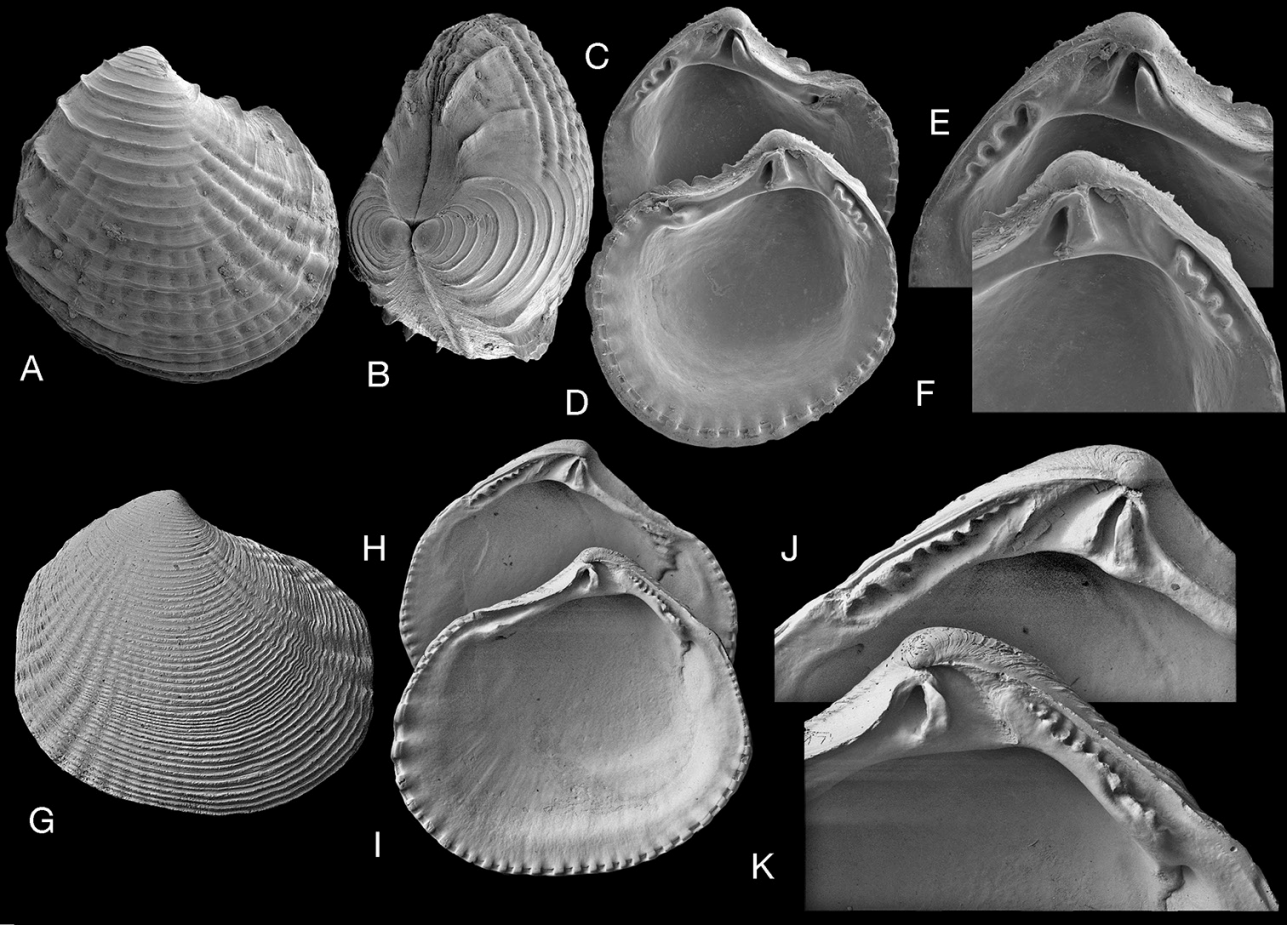
*Pusillolucina pusilla* and *P. denticula*. **A–D***Pusillolucina pusilla* (Glover & Taylor, 2016) **A***Pillucina pusilla* Holotype (MNHN IM-2000-26591) Philippines, Bohol Island, L 1.2 mm **B** holotype dorsal view **C, D***P. pusilla* interior of left and right valves, paratype (MNHN IM-2000-26592), L 1.05 mm **E, F** detail of hinge teeth in **C, D**. **G–K***Pusillolucina denticula* (Glover & Taylor, 2001) Durban Bay, South Africa **G** exterior of right valve, paratype (NMSA), L 2.9 mm **H, I** interior of left and right valves, holotype (NMSA), L 3.5 mm **J, K** detail of hinge teeth.

###### Pusillolucina denticula

Taxon classificationAnimaliaLucinidaLucinidae

(Glover & Taylor, 2001)

805A121F-B73D-596C-9858-301E07C6D88C

Figs 8 G–K

Pillucina denticula Glover & Taylor, 2001: 271, figs 7a–g.

####### Type material.

***Holotype***: NMSA B310/T1758, L 3.5 mm; ***Paratypes***: NMSA V8402/T1759, L 2.9 mm, 2.8 mm, 3.1 mm, NHMUK 20000377, L 3.5 mm.

####### Type locality.

Durban Bay, South Africa.

####### Description.

Shells small, L to 3.5 mm, robust, sub-circular to ovoid in outline. Sculpture of fine, closely spaced, commarginal lamellae crossed by low, rounded radial ribs that are prominent and broader towards anterior and posterior. Ribs inconspicuous in central part of shell. Lunule long, lanceolate. Ligament internal, short. Right valve with a single cardinal tooth, a prominent anterior lateral tooth and a long posterior lateral tooth divided into 8–10 small cusps. Left valve with two cardinal teeth, a small anterior lateral and a posterior tooth divided into sockets for cusps of the RV. Anterior adductor muscle scar short and barely detached from the pallial line. Inner shell margin crenulate, with crenulations more widely spaced anteriorly.

###### Pusillolucina arabica sp. nov.

Taxon classificationAnimaliaLucinidaLucinidae

4CBDD728-1E82-50DC-96E8-AC1BAD8C281D

http://zoobank.org/BD3CBB6A-99A1-4F13-9553-BAC006538A64

Figs 3 , 9 , 10

####### Type material.

***Holotype***: NHMUK 20191077 L 2.4 mm. ***Paratypes***: figured L 2.1 mm, L 2.0 mm, L 1.9 mm, unfigured 11 sh, 2 v (NHMUK 20191078).

####### Type locality.

Arabian Gulf, Tarut Bay, Saudi Arabia, dredged (17.5.1971) K. Smythe collection.

####### Material examined.

NHMUK consultancy report ECM 5027C/06. Arabian Gulf: station 36 FC, 27°42'38"N, 52°11'16"E 25 m (NHMUK20191080), stn 39 FC, 27°42'05"N, 52°10'50"E 31 m, NHMUK 20191081), stn 41 FC, 27°42'31"N, 52°10'02"E 32 m (NHMUK 20191079), stn 48 FC, 27°45'0.27"N, 52°07'46"E 23 m (NHMUK 20191082).

####### Etymology.

*arabica* from Latin *arabicus*. Used as an adjective.

####### Diagnosis.

*Pusillolucina* with posterior lateral teeth divided into four or five cusps and sockets.

####### Description.

Shell very small, L to 2.4 mm, ovate, umbones prominent, sculpture of closely spaced, narrow, commarginal lamellae, sometimes elevated at posterior and anterior dorsal margins, crossed at anterior and posterior by low radial ribs, juvenile shells with commarginal lamellae only. Colour: white, translucent when wet. Protoconch: P1 84 µm, P1 + P2 = 155 µm, P2 with numerous growth increments. Lunule broadly lanceolate, smooth. Ligament internal, short, set alongside cardinal teeth. Hinge: right valve with single cardinal tooth, anterior lateral tooth located above anterior adductor muscle. Posterior lateral tooth long, divided into four or five cusps, left valve with two cardinal teeth, the anterior larger, anterior lateral tooth small, posterior lateral tooth divided into four or five sockets for cusps of right valve. Anterior adductor muscle scar short, barely detached from pallial line, posterior scar ovoid. Inner shell margin crenulate, more strongly to anterior and posterior.

####### Remarks.

*Pusillolucina arabica* differs from the Philippine *P. pusilla* by the more ovate outline, and the less prominent commarginal and radial sculpture. By comparison, *P. africana* has finer more closely spaced commarginal sculpture and more cusps (7–8) on the posterior lateral tooth. From South Africa *P. denticula* has much finer commarginal sculpture and up to 10 smaller cusps on the posterior lateral tooth. From Madagascar *P. biritika* sp. nov. has only three cusps on the posterior lateral tooth.

Despite its small size, *P. arabica* has the characteristic, thick inner ctenidial demibranchs with a well-developed bacteriocyte zone packed with symbiotic bacteria ca 3–4 µm (Fig. 10N, O) similar to other lucinids.

**Figure 9.**
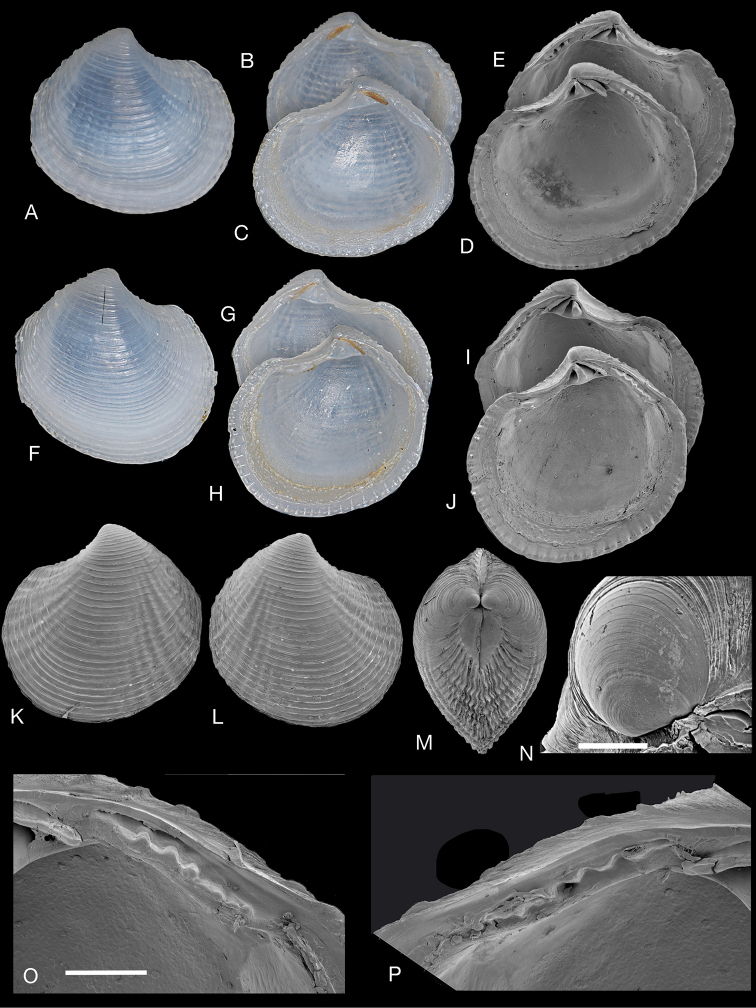
*Pusillolucina arabica* sp. nov. Tarut Bay, Saudi Arabia, Arabian Gulf. **A–C** Holotype (NHMUK 20191077) exterior of right valve and interior of left and right valves, L 2.4 mm **D, E** holotype, interior of left and right valves SEMs **F–H** paratype (NHMUK 20191078) exterior of right valve and interior of left and right valves, L 2.1 mm **I, J** paratype F-H (NHMUK 20191078) interior of left and right valves SEM**K, L** paratype (NHMUK 20191078) exterior of left and right valves, L 2.0 mm **M** paratype (NHMUK 20191078) dorsal view, L 1.9 mm **N** protoconch of holotype, scale bar 50 µm **O, P** paratype **I–J** detail of multi-cuspate posterior lateral hinge teeth. Scale bar: 200 µm.

**Figure 10.**
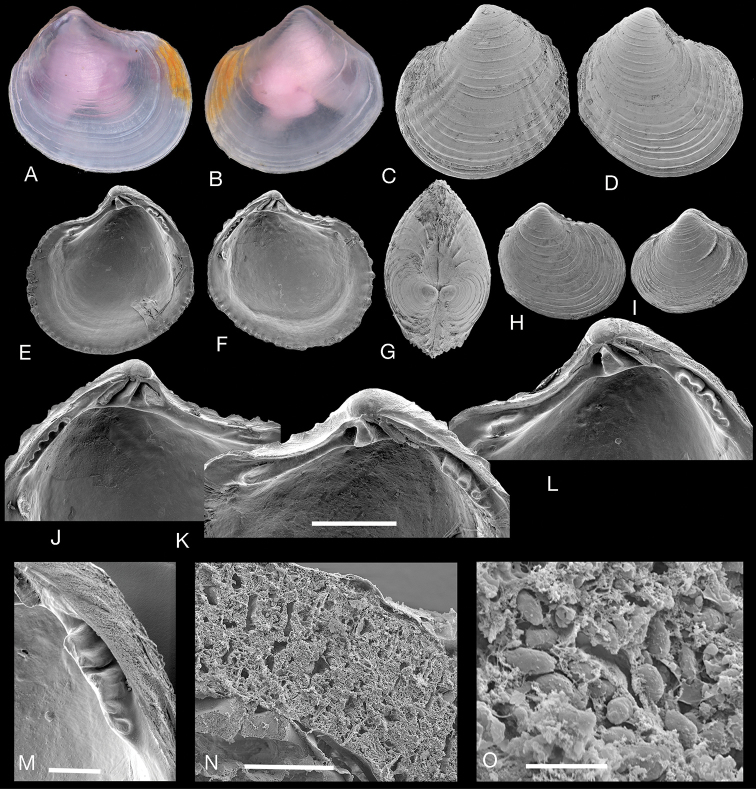
*Pusillolucina arabica* sp. nov. Arabian Gulf NHMUK consultancy samples- see text for details. **A** Lateral view of right valve stn 41C (NHMUK 20191079) stained with Rose Bengal, L 1.3 mm **B** lateral view of left valve stn 41C (NHMUK 20191079), L 1.2 mm **C, D** exterior of left (L 1.5 mm) and right (L 1.3 mm) valves stn 39FC (NHMUK 20191081) **E, F** Interior of right (L 1.4 mm) and left (L 1.5 mm) valves stn 36FC (NHMUK 20191080) **G** dorsal view, stn 39FC (NHMUK 20191081), L 1.3 mm. **H** juvenile shell, stn 48FC (NHMUK 20191082), L 1.1 mm **I** juvenile shell, stn 36FC (NHMUK 20191080), L 0.9 mm **J–L** hinge teeth of left and right valves stn 36FC (NHMUK 20191080) **M** detail of posterior lateral tooth of right valve stn 36FC (NHMUK 20191080) **N** section through a ctenidial demibranch with thickened bacteriocyte zone, critical point dried preparation **O** symbiotic bacteria. Scale bars: 300 µm (**J–L**); 100 µm (**M**); 50 µm (**N**); 4 µm (**O**).

###### Pusillolucina africana sp. nov.

Taxon classificationAnimaliaLucinidaLucinidae

48D77C5A-B0D2-57DA-8130-C6D3C10CDDA2

http://zoobank.org/2533482F-2D76-413A-AB4E-1AC96BA998C9

Fig. 11

####### Type material.

***Holotype***: MNHN-IM-2000-35107, sh, L 2.3 mm; ***Paratypes***: 10 v, L 1.9–2.2 mm MNHN-IM-2000-35108, 3 v NHMUK 20191083.

####### Type locality.

Mozambique, Inhaca Island, Baia Campessuane, 3–4 m, INHACA stn MD1, 26°03.6'S, 32°56.6'E. 25NOV2011.

####### Etymology.

Named for Africa, used as an adjective.

####### Diagnosis.

*Pusillolucina* with posterior lateral teeth divided into seven or eight cusps and sockets.

####### Description.

Shell very small, L to 2.4 mm, ovate, umbones prominent, sculpture of closely spaced, narrow, commarginal lamellae, sometimes slightly elevated at posterior and anterior dorsal margins, crossed at anterior and posterior by low radial ribs, juvenile shells with commarginal lamellae only. Protoconch: P1 ca 75 µm, P1 + P2 = 140 µm, P2 with numerous growth increments. Lunule long, broadly lanceolate, smooth. Ligament internal, short, set on triangular resilifer alongside cardinal teeth. Hinge: right valve with single cardinal tooth, anterior lateral tooth located above anterior adductor muscle. Posterior lateral tooth long, divided into seven or eight cusps, left valve with two cardinal teeth, the anterior larger, anterior lateral tooth small, posterior lateral tooth with seven or eight sockets for cusps of right valve. Anterior adductor muscle scar short, barely detached from pallial line, posterior scar ovoid. Pallial line continuous. Inner shell margin crenulate, more strongly to anterior.

####### Remarks.

For comparison with other species see *P. arabica* above.

**Figure 11.**
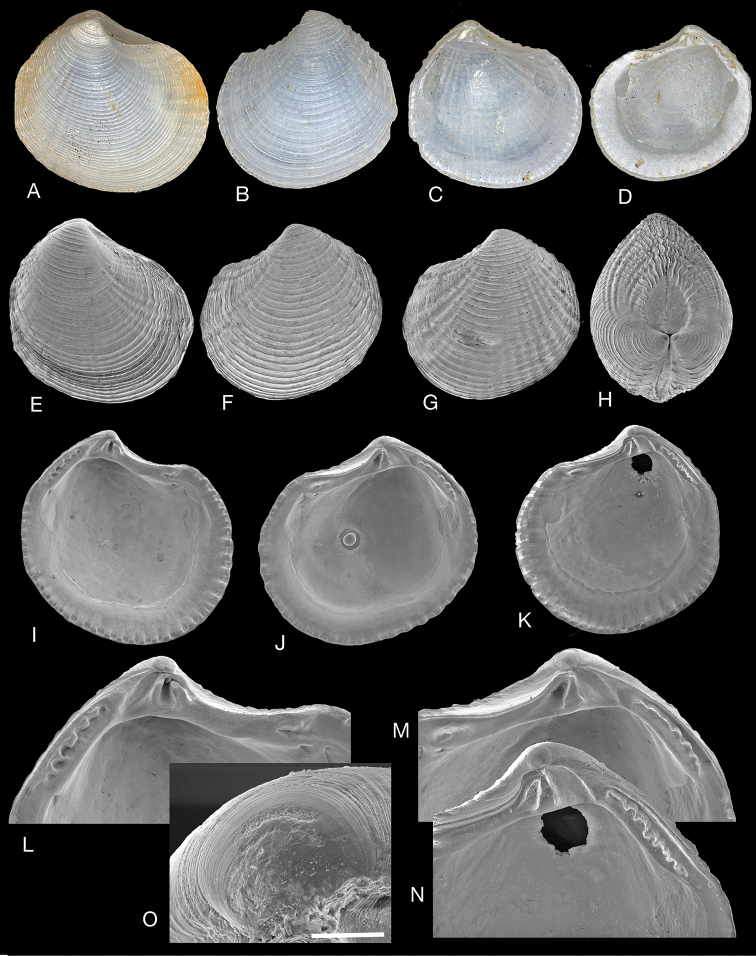
*Pusillolucina africana* sp. nov. Mozambique, Inhaca Island, Baia Campessuane, 3–4 m, INHACA stn MD1. **B–O** Paratypes (MNHN-IM-2000-35108). **A** Holotype (MNHN-IM-2000-35107), L 2.3 mm **B** paratype exterior left valve, L 2.1 mm **C** interior of left valve, L 2.1 mm **D** interior right valve, L 1.9 mm **E** exterior right valve, L 2.2 mm **F** exterior left valve, L 2.2 mm **G** exterior of left valve, L 2.2 mm **H** dorsal view, L 2.4 mm **I** interior of left valve, L 2.1 mm **J** interior of right valve, L 2.1 mm **K** interior of right valve, L 2.1 mm **L–N** detail of hinges of **I, J, K**. **O** Protoconch. Scale bar: 50 µm (**O**).

###### Pusillolucina biritika sp. nov.

Taxon classificationAnimaliaLucinidaLucinidae

8134EE37-2602-5F60-AD98-23D16336A64C

http://zoobank.org/FBD2E714-389D-4B91-BE1A-8BCB5820ACE9

Fig. 12

####### Type material.

***Holotype***: MNHN-IM-2000-35109 1.1 mm, ***Paratypes***: MNHN-IM-2000-35110, MNHN-IM-2000-35111, MNHN-IM-2000-35181, NHMUK 20191084 lengths 1.1–1.5 mm.

####### Type locality.

***Holotype***: South Madagascar east of Cap Antsirabe, 49–52 m, ATIMO-VATAE stn TP11, 25°02.8'S, 47°01.3'E. 06MAY2010 (MNHN-IM-2000-35109).

***Paratypes***: South Madagascar east of Cap Antsirabe, 49–52 m, ATIMO-VATAE stn TP11, 25°02.8'S, 47°01.3'E. 06MAY2010 (MNHN-IM-2000-35181 5 v).

South Madagascar off Baie Fort-Dauphin 54–56 m, ATIMO-VATAE stn TP18, 25°02.4'S, 47°03.2.6'E, 11MAY2010 (MNHN-IM-2000-35110 6 sh, 9 v. NHMUK 20191084 1 sh, 2 v).

Northwest Madagascar, S of Cap St Sébastien, 42–44 m, MIRIKY stn DW3202, 12°35.6'S, 48°49.9'E, 29JUN2009 (MNHN-IM-2000-35111 6 v).

####### Etymology.

Biritika, meaning extremely small in Malagasy. Used as a noun in apposition.

####### Diagnosis.

*Pusillolucina* with posterior lateral teeth divided into three cusps and sockets.

####### Description.

Very small, L 1.5 mm, sub-ovate, longer than high, umbones prominent. Sculpture of fine commarginal lamellae crossed at anterior and posterior by low, rounded, radial ribs, juvenile shells with commarginal lamellae only. Some lamellae extended as scales along posterior dorsal margin. Lunule long, lanceolate. Ligament short internal on short resilifer. Hinge: RV with single cardinal tooth and single anterior lateral tooth, posterior lateral tooth short, divided into three cusps, LV with two cardinal teeth the posterior-most thin, posterior lateral tooth with three sockets for cusps of the right valve. Anterior adductor muscle scar short, barely detached from pallial line, posterior scar ovate. Pallial line entire. Shell margin coarsely crenulate, more strongly to anterior and posterior.

**Figure 12.**
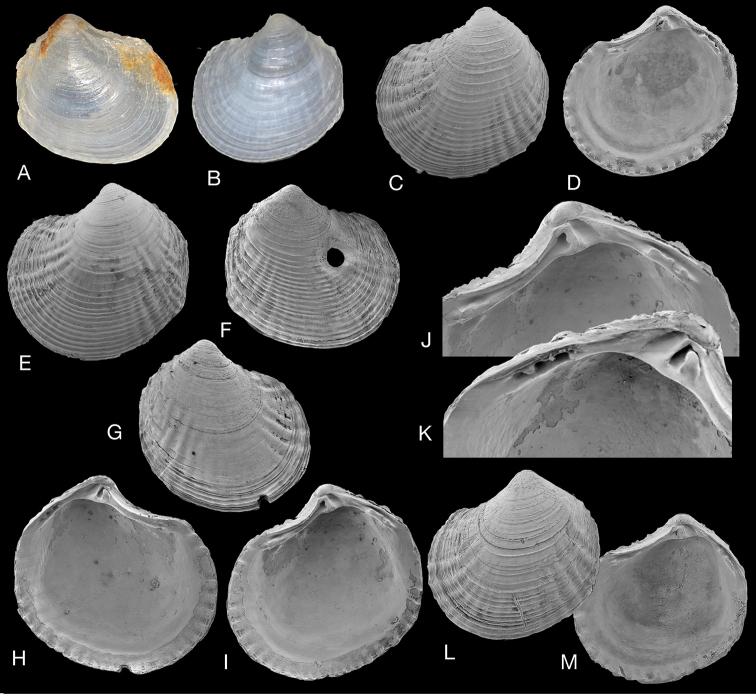
*Pusillolucina biritika* sp. nov. **A, B** South Madagascar east of Cap Antsirabe, 49–52 m, ATIMO-VATAE stn TP11, 25°02.8'S, 47°01.3'E. (MNHN) **C–E, L, M** South Madagascar off Baie Fort-Dauphin 54–56 m, ATIMO-VATAE stn TP18, 25°02.45'S, 47°03.26'E (MNHN) **F–K** Northwest Madagascar, S of Cap St Sébastien, 42–44 m, MIRIKY stn DW3202, 12°35.6'S, 48°49.9'E (MNHN). **A** Holotype, stn TP11 (MNHN-IM-2000-35109), L 1.1 mm **B** paratype stn TP11 (MNHN-IM-2000-35181) L 1.5 mm **C** paratype stn TP18 (MNHN-IM-2000-35110) exterior of left valve, L 1.3 mm **D** paratype stn TP18 (MNHN-IM-2000-35110) interior of right valve, L 1.3 mm **E** paratype stn TP18 (MNHN-IM-2000-35110) exterior of left valve L 1.5 mm **F** paratype stn DW3202 (MNHN-IM-2000-35111). exterior of right valve L 1.4 mm **G** paratype stn DW3202 (MNHN-IM-2000-35111). exterior of right valve L 1.1 mm **H** paratype stn DW3202 (MNHN-IM-2000-35111) interior of left valve L1.4 mm **I** paratype stn DW3202 (MNHN-IM-2000-35111) interior of right valve L 1.3 mm **J, K** detail of hinge teeth of **H** and **I**. **L, M** Paratype TP18 (MNHN-IM-2000-35110) exterior of left valve and interior of right valve L 1.2 mm.

###### Notocina gen. nov.

Taxon classificationAnimaliaLucinidaLucinidae

762A9A23-09E3-5D41-AEDD-0D89BBD4B913

http://zoobank.org/50070A53-D987-4091-958E-35BB99DE91A4

####### Type species.

*Epicodakia falklandica* Dell, 1964. Here designated.

####### Diagnosis.

Small (L to 3 mm), sub-ovoid, slightly longer than high. Umbones prominent. Posterior dorsal margin straight. Sculpture of low, rounded, commarginal lamellae with poorly defined fine radial ribs to anterior and posterior. Microsculpture densely punctate. Ligament short, protrudes above dorsal margin. Lunule broadly lanceolate. Hinge line narrow, left valve with two small cardinal teeth and anterior and posterior lateral teeth. Right valve with single, slightly bifid cardinal tooth and anterior and posterior lateral teeth. Anterior adductor muscle scar short, slightly detached from pallial line, inner shell margin finely denticulate.

####### Etymology.

*notos* in Greek meaning south, -*cina* as an abbreviation of *Lucina*. In reference to the southern Atlantic distribution. Female gender.

####### Remarks.

Dell (1964) placed *N. falklandica* in *Epicodakia* (type species *E. consettiana* Iredale, 1930) because of some similarity of hinge teeth and sculpture. He remarked that only two species of *Epicodakia* were known (*E. consettiana* Iredale, 1930 and *E. neozelanica* Powell, 1937) but since then a number of other species from the Indo-West Pacific, Western Atlantic and Eastern Pacific have been classified in the genus (Glover and Taylor 2007; 2016; Taylor et al. 2016; MolluscaBase). Molecular analyses place *Epicodakia* in the subfamily Codakiinae, close to *Ctena* species (Taylor et al. 2016). Inclusion of ‘Epicodakia’ falklandica indicates it belongs in the subfamily Lucininae in a well-supported position as a sister species to *Troendleina suluensis* Glover & Taylor, 2106 from 150–600 m in the Sulu Sea. *Troendleina* includes three described species, all from deeper water; *T. marquesana* Cosel & Bouchet, 2008 from the Marquesas Islands, *T. musculator* Cosel & Bouchet, 2008 from the Solomon Islands and *T. suluensis* Glover & Taylor, 2016 from the Philippines and we are aware of further undescribed species. There is no close similarity of *Troendleina* to *E. falklandica* in shell characters: species of the former are larger with lengths of 30–40 mm, a sculpture of growth increments with fine radial threads, small cardinal teeth, obscure or absent lateral teeth, and a finely dentate inner shell margin. *Notocina falklandica* has some similarity to *Parvilucina* species in size and shell characters but is not closely aligned in molecular trees. Another unusual small species *Guyanella clenchi* from the southern Caribbean (Taylor et al. 2016) is similar to *Notocina falklandica* in size but differs in having a distinctive internal ligament and lacks any radial sculpture. Neither *Troendleina*, *Parvilucina* nor *Guyanella* species possess the punctate microsculpture that occurs in *N. falklandica* and sporadically amongst other Lucinidae including species of Myrteinae, some *Ctena*, *Epicodakia* and *Codakia* species (Codakiinae) and *Funafutia levukana* (Lucininae) (Glover and Taylor 2016).

###### Notocina falklandica

Taxon classificationAnimaliaLucinidaLucinidae

(Dell, 1964)

1B041ECE-BBED-5C03-B853-0A7AC729E9EE

Fig. 13

Epicodakia falklandica Dell, 1964: 206, fig. 4 (17, 18, 19).

####### Type material.

***Holotype***: NHMUK 1962857/1; ***Paratypes***: NHMUK 1962858/1; 1962859/1; 1962860/1; 1962861/2; 1962862/2.

####### Type locality.

Off Falkland Islands, Discovery station WS 766. 44°58'S, 60°05'30"W, 545 m. Paratype depth range 105–219 m.

####### Material examined.

South Georgia, 53.561108S, 37.88494W, 221 m BIOPEARL cruise 1, 05.04.2006 sample B-06-1167. GenBank numbers: 18S KF741615, 28S KF741644, cyt b KF741675.

####### Description.

**Figure 13.**
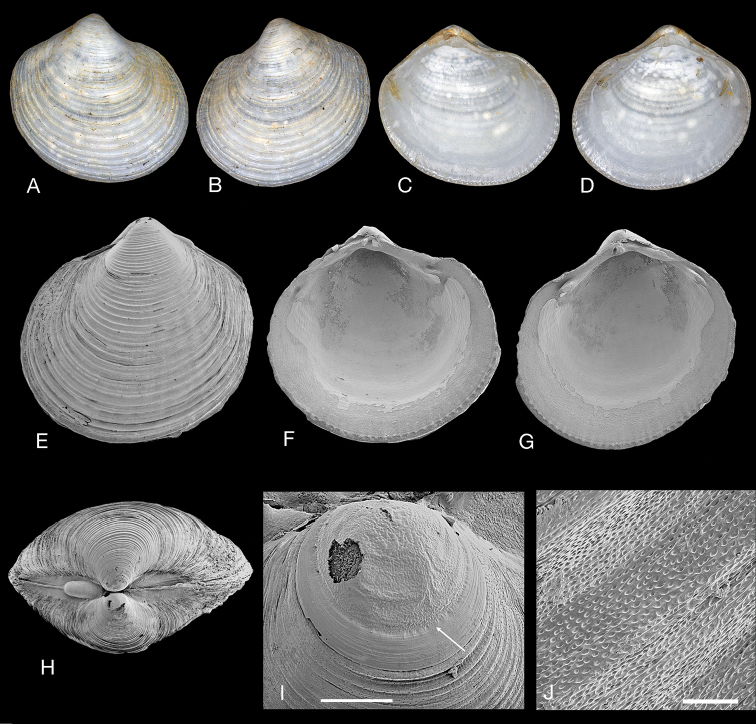
*Notocina falklandica* (Dell, 1964). **A–D** Holotype of *Epicodakia falklandica* (NHMUK 1962857/1) exterior and interior views of both valves, L 2.5 mm **E–J***N. falklandica* paratype (NHMUK 1962858) Falkland Islands, Discovery stn WS 214, 208 m **E** exterior of left valve, L 1.9 mm **F, G** interior of left and right valves, L 2.3 mm **H** dorsal view, L 1.9 mm **I** protoconch, arrow indicates boundary of P1 and P2**J** detail of **E** showing punctate microsculpture. Scale bar: 100 µm (**I**); 20 µm (**J)**.

Shells small, L 2–3 mm, ovoid, slightly longer than high. Umbones prominent. Posterior dorsal margin straight. Sculpture of low rounded commarginal lamellae with narrow interspaces. Faint radial ribs visible to anterior and posterior. Microsculpture of dense fine punctae (2–3 µm in diameter). Protoconch: P1 = 188 µm, P1 + P2 = 242 µm, P2 with fine growth increments (South Georgia shell). Ligament short, protruding above posterior dorsal margin (Fig. 13J), set in shallow nymph. Escutcheon well defined, smooth. Lunule slightly impressed, broadly lanceolate. Hinge line narrow, left valve with two small cardinal teeth and anterior and posterior lateral teeth. Right valve with single, slightly bifid cardinal tooth and anterior and posterior lateral teeth. Anterior adductor muscle scar short, barely detached from pallial line, posterior adductor scar ovoid. Pallial line entire. Inner shell margin finely denticulate.

####### Distribution.

**South Atlantic**: around Falkland Islands (Dell 1964), Argentina, outer continental shelf off Buenos Aires (Roux et al. 1993); Patagonia shelf, South Georgia, South Orkney Islands (Zelaya 2005). Depth range 100–545 m (distribution map at https://www.gbif.org/species/6523387).

## Discussion

There is a general perception that bivalves having chemosymbiosis with thiotrophic or methanotrophic bacteria are large, as exemplified by Vesicomyidae (*Calyptogena magnifica* Boss & Turner, 1980, 263 mm), Modiolinae (*Bathymodiolus boomerang* Cosel amp; Olu, 1998, 360 mm), Solemyidae (*Acharax bartschi* (Dall, 1908), 210 mm) that live at hydrothermal vents and hydrocarbon seeps. Among the several bivalve families with chemosymbiotic life habits Lucinidae is by far the most diverse with more than 400 species occupying a wide range of habitats from the intertidal to bathyal depths and spanning a wide size range from 1.5 to 170 mm. The largest living species are *Meganodontia acetabulum* Bouchet & Cosel, 2004 with shell lengths up to 170 mm and *Codakia distinguenda* (Tryon, 1872) at 160 mm, while the Eocene fossil, *Superlucina megameris* (Dall, 1901), attained a shell height of 310 mm (Taylor and Glover 2009). Nevertheless, most species of Lucinidae are much smaller, with recent studies revealing minute species as small as 1.5 mm (Glover and Taylor 2016, Taylor et al. 2016). Additionally, with availability of molecular phylogenies, paraphyly is increasingly being recognised in genera and families previously classified by morphological characters. Such paraphyly has been found in the small lucinids previously assigned to *Pillucina*; one group consists of several species similar to the genotype of *Pillucina* and the other includes species some previously neglected but now assigned to the new genera, *Rugalucina* and *Pusillolucina*, that are also distinctive in morphology.

One of the foci of this paper has been the recognition of some very small lucinid species within the new genus *Pusillolucina* with adult shell lengths of 1–3 mm. All the species in this genus have a very unusual multi-cuspate lateral dentition not seen in any other lucinids. The recognition of these minute lucinids extends the morphological and functional range of Lucinidae. Even the smallest have chemosymbiosis with thickened ctenidial demibranchs occupied by symbiotic bacteria. Other minute lucinids include *Guyanella clenchi* (Altena, 1968) 1–2 mm and *Parvilucina latens* Taylor & Glover, 2016, 2–3 mm, both documented from Guadeloupe but likely occur more widely in the western Atlantic (Taylor et al. 2016). The latter species was first detected in molecular analyses having initially been confounded with *Parvilucina pectinella* (C.B. Adams, 1852). Additionally, most species of *Liralucina*, have shell lengths less than 5 mm (Glover and Taylor 2007), they are little recorded but common in some coral reef associated habitats (Glover and Taylor 2007).

Another small species that has been phylogenetically misplaced is *Notocina falklandica* widely recorded from outer shelf and bathyal depths around the Falkland Islands, Argentina and Uruguay. Since the original description by Dell (1964) its placement in *Epicodakia* (Codakiinae) had been problematic but molecular data now shows that it should be classified in the subfamily Lucininae in a monospecific genus close to *Troendleina* from the Indo-West Pacific with no comparable taxa known from the southern Atlantic.

Two species described here, *Rugalucina angela* and ‘R.’ cracentis, are abundant in the northern Red Sea where a surprising number of other lucinids have been recorded. From intensive sampling around Safaga Bay (Zuschin and Oliver 2003; 2005) lucinids were amongst the most abundant bivalves with 15 species comprising nearly 40 % of the shells recovered, most frequently *Cardiolucina semperiana* (Issel, 1869), *Wallucina erythraea* (Issel, 1869) and ‘*Pillucina fischeriana*’. With taxonomic refinements 19 species are now recognised from the Safaga area (Taylor and Glover 2005, herein). The northern Red Sea is a highly oligotrophic environment and, with a nutritional strategy of chemosymbiosis utilising sulphides from the benthic substrate, lucinids can survive in nutrient poor habitats where food availability for suspension feeding bivalves is limited.

## Supplementary Material

XML Treatment for Rugalucina

XML Treatment for Rugalucina angela

XML Treatment for Rugalucina vietnamica

XML Treatment for Rugalucina munda

XML Treatment for ‘Rugalucina’ cypselis

XML Treatment for ‘Rugalucina’ cracentis

XML Treatment for Pusillolucina

XML Treatment for Pusillolucina pusilla

XML Treatment for Pusillolucina denticula

XML Treatment for Pusillolucina arabica

XML Treatment for Pusillolucina africana

XML Treatment for Pusillolucina biritika

XML Treatment for Notocina

XML Treatment for Notocina falklandica
